# Itaconate as a key regulator of respiratory disease

**DOI:** 10.1093/cei/uxad127

**Published:** 2023-11-29

**Authors:** Christina Michalaki, Gesa J Albers, Adam J Byrne

**Affiliations:** National Heart and Lung Institute, Imperial College London, London, SW7 2AZ, UK; National Heart and Lung Institute, Imperial College London, London, SW7 2AZ, UK; National Heart and Lung Institute, Imperial College London, London, SW7 2AZ, UK; School of Medicine and Conway Institute of Biomedical Sciences, University College Dublin, Belfield, Dublin 4, Ireland

**Keywords:** Respiratory disease, itaconate, macrophages

## Abstract

Macrophage activation results in the accumulation of endogenous metabolites capable of adopting immunomodulatory roles; one such bioactive metabolite is itaconate. After macrophage stimulation, the TCA-cycle intermediate *cis*-aconitate is converted to itaconate (by aconitate decarboxylase-1, ACOD1) in the mitochondrial matrix. Recent studies have highlighted the potential of targeting itaconate as a therapeutic strategy for lung diseases such as asthma, idiopathic pulmonary fibrosis (IPF), and respiratory infections. This review aims to bring together evidence which highlights a role for itaconate in chronic lung diseases (such as asthma and pulmonary fibrosis) and respiratory infections (such as SARS-CoV-2, influenza and *Mycobacterium tuberculosis* infection). A better understanding of the role of itaconate in lung disease could pave the way for novel therapeutic interventions and improve patient outcomes in respiratory disorders.

## Teaser

Recent studies have highlighted the potential of targeting itaconate as a therapeutic strategy for lung diseases such as asthma, idiopathic pulmonary fibrosis (IPF), and respiratory infections. This short review aims to bring together evidence which highlights a role for itaconate in chronic lung disease. A better understanding of the role of itaconate in respiratory disorders could pave the way for novel therapeutic interventions.

## The metabolic environment of the lungs

The lung is one of the most metabolically active organs of the human body and is responsible for maintaining optimal gas exchange [1, 2]. Thus, local structural, as well as immune cells, is required to maintain homeostasis while facilitating gas exchange [1]. As a consequence, cells are optimally adapted to the pulmonary niche and are highly specialized, resulting in high-energy demands and low pulmonary glucose concentrations [3, 4]. Interestingly, the majority of glucose is metabolized into lactate as opposed to CO_2_, indicative of a high rate of glycolysis in the lung [4, 5]. Under anaerobic conditions, the conversion of glucose into lactate is common; however, when this phenomenon occurs during aerobic conditions it is termed as the Warburg effect [5, 6]. It is hypothesized that the lung adapted to favour aerobic glycolysis to reduce the local oxygen consumption [2]. Subsequently, more oxygen can be transported to other tissues [2]. Moreover, low-glucose concentrations in the airways might contribute to airway defence against infection by limiting the availability of nutrients thereby restricting bacterial growth [7]. Recently, there has been a new focus on how the unique metabolic environment of the respiratory tract shapes local immune responses.

The most abundant immune cells in the healthy lung are airway macrophages (AMs) which are strategically located at the interface between the internal and external pulmonary environment [8–10]. It has recently become clear that immune cell responses are underpinned by cellular metabolism. Activation of macrophages via toll-like receptors (TLRs) leads to increased glycolytic activity and to the accumulation of different immunomodulatory metabolites, such as itaconate [11–14]. As a consequence of a broken TCA cycle at the isocitrate dehydrogenase in glycolytic macrophages, the metabolic flux is redirected and favours the production of citrate rather than α-ketoglutarate [11–14]. Subsequently, citrate accumulates and is fed into the synthesis of itaconate [11–14]. Aconitate decarboxylase 1 (*Acod1*), also known as immune-responsive gene 1 (*Irg1*), is the gene encoding the enzyme that facilitates itaconate production through the decarboxylation of the TCA cycle intermediate, *cis*-aconitate [13]. Itaconate accumulates within and is secreted by activated macrophages [15, 16] and can drive immune responses in surrounding cells via activation of oxoglutarate receptor 1 (OXGR1) [15]. While itaconate can finetune the cellular metabolism of macrophages via the inhibition of succinate dehydrogenase (SDH), it has additionally been shown to exhibit multiple immunomodulatory functions. Chronic diseases and pulmonary infections in the lung, where substantial evidence has been accumulated linking itaconate with disease, will be focused upon; other disease area where less is known about the role of itaconate (e.g. Chronic obstructive pulmonary disease, COPD) is beyond the scope of this mini-review.

## Anti-inflammatory functions of itaconate

**Figure 1:**
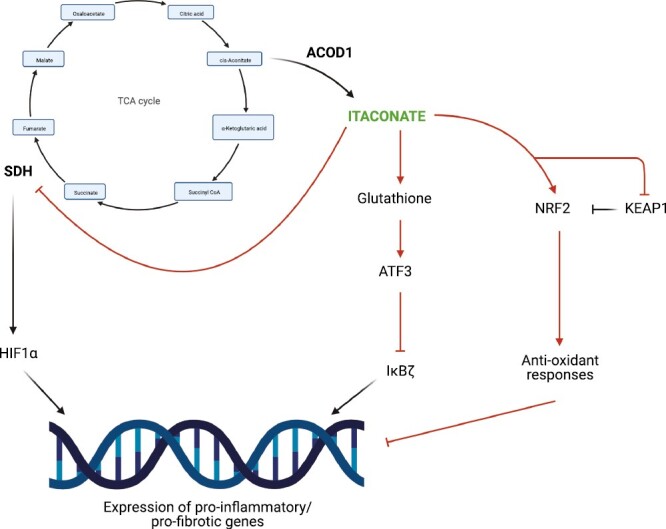
Anti-inflammatory functions of itaconate. The immunomodulatory metabolite itaconate can exhibit anti-inflammatory properties via different pathways. Itaconate is produced by cis-aconitate when the TCA cycle is disturbed. The enzyme cis-aconitate decarboxylase 1 encoded by the highly expressed Acod1 (also known as Irg1) in airway macrophages during inflammatory conditions facilitates itaconate production. Itaconate has been shown to have weak inhibitory effects of TCA cycle enzyme succinate dehydrogenase (SDH) which limits the stabilisation of HIF1α and thereby inhibits the transcription of pro-inflammatory genes. Moreover, itaconate can modify glutathione, leading to the accumulation of ATF3, which inhibits IκBζ, a driver of pro-inflammatory responses. Thirdly, itaconate alkylates cysteine residues on KEAP1 and thus, stabilizes NFR2 to promote anti-oxidant responses inhibiting the expression of pro-inflammatory genes (created with BioRender.com).

Itaconate and its derivates may act as anti-inflammatory mediators in multiple ways, such as through direct modification of proteins (Fig. 1). One way through which itaconate can exert its anti-inflammatory effects is through reversibly and competitively inhibiting SDH, resulting in the intracellular accumulation of succinate [12, 17, 18]. This inhibition limits hypoxia-inducible factor 1-alpha (HIF1α) stabilization thereby preventing the transcription of pro-inflammatory genes [12]. Another way through which itaconate can activate anti-inflammatory pathways is by stabilizing nuclear factor erythroid 2-related factor 2 (NRF2) [14]. During basal conditions, Kelch-like ECH-associated protein 1 (KEAP1) forms a complex with NRF2, marking NRF2 for degradation and preventing it from migrating into the nucleus. Using its electrophilic α,β-unsaturated carboxylic acid, itaconate can alkylate cysteine residues on KEAP1 [14]. Upon alkylation of KEAP1 by itaconate, NRF2 is released from the complex and translocates into the nucleus where it can drive the expression of anti-oxidant genes, such as *Hmox1*, *Nqo1* and *GclmI* [19, 20]. Ultimately, this results in reduced type I interferon responses, as well as pro-inflammatory and pro-fibrotic gene expression [14]. However, the role of itaconate in activating NRF2 is complex, and it could be context dependent. In hepatocytes, itaconate-mediated activation of NRF2 protected against liver ischaemia-reperfusion injury, thereby indicating an important role of itaconate in non-immune cells [21]. Sun et al. also demonstrated that the effects of itaconate and NRF2 activation may be stimulus-dependent and may depend on the origin of itaconate (endogenous or exogenous) [22]. Endogenous itaconate did not activate NRF2 in macrophages during particulate matter (PM)-induced inflammation [22]. Conversely, 4-octyl itaconate (4-OI) administration attenuated PM-induced inflammation and activated NRF2. However, NRF2 activation was not necessary for the anti-inflammatory effects of 4-OI [22].

In addition, itaconate can exhibit anti-inflammatory functions via NRF2-independent pathways. In the context of neuropathic pain, itaconate has been shown to promote IL-10 production [23]. LPS can induce the expression of *Tnf* and *Nfkbiz* via activation of TLRs independent of NRF2 [24]. Subsequently, IκBζ, encoded by *Nfkbiz*, enables the transcription of *Il6* and *Il12* [25]. However, via inhibition of IκBζ, dimethyl itaconate (DI) can block the transcription of IκBζ-target genes by modifying glutathione and thus, upregulating activating transcription factor 3 (ATF3) which in turn leads to a reduced production of pro-inflammatory cytokines [26].

## Itaconate in allergic airway disease

**Figure 2.**
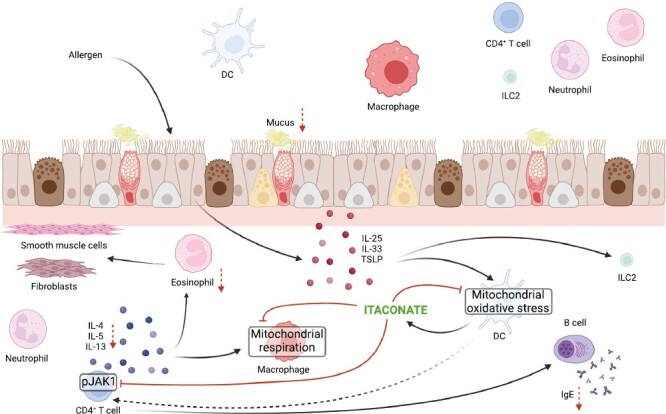
Itaconate in allergic airway disease. In murine models of asthma, itaconate is produced by lung DCs following allergen exposure. Subsequently, itaconate can reduce mitochondrial stress in DCs as well as act on surrounding immune cells. Moreover, itaconate inhibits alternative activation of macrophages and mitochondrial respiration in macrophages. Itaconate inhibits the phosphorylation of JAK1 in CD4^+^ T cells which results in a reduction in type 2 cytokine production, limiting the recruitment of eosinophils and class switching of B cells and IgE secretion. Finally, this diminishes mucus production and airway hyper-responsiveness (created with BioRender.com).

Allergic airway disease (AAD), such as asthma is characterised by inflammation and remodelling of the airways, augmented production of mucus and airway hyperresponsiveness (AHR), resulting in a decline in lung function [27]. Asthma can be broadly differentiated into type 2 (T2) and non-T2 asthma and patients with T2 asthma show increased numbers of eosinophils, T helper 2 cells (TH2), innate lymphoid cells 2 (ILC2) and augmented secretion of interleukin (IL)-4, IL-5, and IL-13 into the airways as well as increased levels of immunoglobulin E (IgE) in the serum [27–30], (Fig. 2). Typically, T2 asthma is triggered by an allergen, such as house dust mite (HDM), and begins during childhood [31]. Allergens contain proteases which cleave tight junctions between pulmonary epithelial cells and thereby compromise the barrier integrity of the epithelium, leading to the production of thymic stromal lymphopoietin (TSLP), IL-33, and IL-25 [32–34]. The release of these alarmins drives the production of T2 cytokines IL-4, IL-5, and IL-13 by CD4^+^ T cells which promotes IgE production by B cells and recruitment of eosinophils [35]. Ultimately, these processes result in airway remodelling and AHR [36–41].

In a murine model of asthma, *Acod1* expression was highly elevated in lung tissue and dendritic cells (DCs) isolated from the lungs of mice sensitised to and challenged with aeroallergen HDM, indicative of an important role for itaconate in AAD [42]. Indeed, *Acod1*-deficiency resulted in worsened disease pathology, characterised by increased eosinophilia, T2 cytokine production, mucus secretion and antibody production [42]. Moreover, the administration of 4-OI limited AAD in HDM- as well as ovalbumin (OVA)-induced models of AAD [42, 43]. While 4-OI limited mitochondrial respiration in macrophages and mitochondrial stress in DCs, it furthermore reduced IL-5 and IL-13 levels via inhibition of the phosphorylation of STAT1 in CD4^+^ T cells [42, 43].

Together, these studies on murine models of asthma suggest that allergen induces the production of itaconate by pulmonary DCs which then acts intracellularly, as well as on surrounding immune cells. Itaconate secreted into the airways inhibits the phosphorylation of STAT1 in CD4^+^ T cells. Thus, the production of T2 cytokines, such as IL-4, IL-5, and IL-13 is diminished. In consequence, T2 cytokine-mediated recruitment of eosinophils, B class switching and production of IgE and alternative activation of macrophages are reduced. Moreover, itaconate directly alters the metabolism of DCs by reducing mitochondrial oxidative stress. In addition, mitochondrial respiration is inhibited in alternatively activated macrophages in response to itaconate, possibly via inhibitory effects of itaconate on SDH. Currently, it is not clear whether itaconate plays a predominant role on T2 or non-T2 asthma. The majority of evidence thus far has been derived from murine models; therefore human studies are required to further understand the role of itaconate in asthma pathogenesis.

## Itaconate in pulmonary fibrosis

Patients with idiopathic pulmonary fibrosis (IPF) showed decreased expression of *ACOD1* in their airway macrophages and reduced levels of itaconate in their bronchoalveolar lavage [44]. In the bleomycin mouse model of pulmonary fibrosis, *Acod1* and itaconate were highly expressed. *Acod1* deficient mice showed increased disease severity compared to wild-type controls, indicated by worsened dynamic resistance, compliance, and elastance [44]. *Acod1*^*−/−*^ mice had increased levels of hydroxyproline and superoxide in their lungs and increased Ashcroft scoring. Adoptive transfer of wild-type-monocyte-recruited AMs in *Acod1*^*−/−*^ mice rescued disease phenotype [44]. Therefore, itaconate is a key regulator of pulmonary fibrosis.

It is known that IPF results from repeated micro-injuries to the alveolar epithelium. Alveolar injury may arise from various factors, such as dust, pollution, fibres, and respiratory infections [45, 46]. Fibrotic-like patterns appeared in 72% of patients requiring mechanical ventilation and in 20% of those who did not, 4 months post-hospitalization in a study by McGroder et al. [47]. Persistent lung abnormalities at 6 months after hospitalization were observed in 20% of the patients in another study. These patients were mostly older males with longer hospital stay [48]. Hence, it is possible that SARS-CoV-2 can result in fibrosis. However, it is not yet known whether this is progressive fibrosis that does not resolve. Indeed, some itaconate derivatives have been shown to have anti-fibrotic effects on other organs, such as the kidneys [49]. 4-OI-induced renoprotection in a unilateral ureteral occlusion model and adenine-induced fibrosis model in rats. As a cell permeable itaconate derivative, 4-OI ameliorated renal fibrosis, by suppressing the expression of transforming growth factor-β (TGF-β)/Smad and nuclear factor kappa B (NF-κB) pathways [49]. Itaconate has also been shown to be protective in liver fibrosis, suggesting broad anti-fibrotic activity outside the lung [21].

## Itaconate in respiratory infections

Acute respiratory infections are caused by bacteria or viruses, they can lead to exacerbation of chronic disease, and are associated with high rates of mortality. Itaconate and its derivatives have been shown to exhibit anti-viral properties in different viral infections such as influenza A virus (IAV) and SARS-CoV-2 infection [50, 51]. In biopsies of COVID-19 patients, the NRF2-itaconate axis was altered and NRF2 expression and antioxidant responses were reduced in COVID-19 patient samples [50]. Consistent with this, the itaconate derivative 4-OI and dimethyl fumarate inhibited intracellular SARS-CoV-2 replication and the expression of inflammatory genes that drive virus-induced pathology [50]. Both 4-OI and itaconate limited IAV replication in PBMCs and human lung cells and DI reduced pulmonary inflammation as well as mortality in a murine model of IAV infection [51]. Recently, mesaconate and citraconate, two naturally occurring isomers of itaconate, were shown to have anti-viral properties by inhibiting the release of new viral particles in influenza-infected A549 cells, thus reducing the expression of *CXCL10* (downstream effector of type I interferon signalling pathway) mRNA and protein [52]. Together, these studies demonstrate a potential for itaconate and its derivatives as an anti-viral treatment, and indeed the anti-inflammatory properties of 4-OI and dimethyl fumarate can be extended to other viruses such as Herpes simplex virus-1 and -2 (HSV-1 and HSV-2), vaccinia virus and Zika virus [50]. Moreover, large-scale screening of a chemical library containing compounds against IAV revealed an itaconate derivative with anti-IAV activity. Subsequently, 25 itaconic acid derivatives were synthesized and their effects against IAV were evaluated *in vitro* [53]. Some of these compounds elicited anti-viral effects by targeting IAV nucleoproteins directly and blocking the export of ribonucleoprotein from the nucleus to the cytosol, thereby limiting viral replication [53]. Two compounds were found to decrease IAV replication and one of them was deemed a promising candidate against IAV that should be further investigated [53].

Besides itaconate exhibiting anti-viral functions, there has been some evidence of itaconate preventing bacterial growth. Itaconate inhibits the activity of isocitrate lyase, an enzyme of the glyoxylate shunt, which is crucial for bacterial growth under certain conditions [13]. Michelucci et al. showed that the growth of bacteria expressing this enzyme, such as *Salmonella enterica* and *Mycobacterium tuberculosis,* was inhibited by itaconate [13]. Itaconyl-CoA, a product of itaconate metabolism, was demonstrated to restrict the growth of *M. tuberculosis*, the pathogen that causes human tuberculosis [54]. However, a study by Wang et al. revealed that *M. tuberculosis* Rv2498c, a bifunctional enzyme, leaded to the dissimilation of itaconate, therefore rendering *M. tuberculosis* resistant to itaconate [55]. In a mouse model of *M. tuberculosis*, itaconate restricted the recruitment of neutrophils and decreased chemokine production in macrophages, thereby preventing pathogen-associated immunopathology [56]. Additionally, neutrophil function impairment has also been implicated in *Staphylococcus aureus* infection. In this disease setting, itaconate inhibited glycolysis and the oxidative burst of neutrophils, which decreased neutrophil survival and their bacteria killing function [57]. Moreover, during infection with *Pseudomonas aeruginosa* itaconate accumulated in the airways and promoted bacterial clearance [15, 58]. Itaconate production by *ACOD1* in *Legionella*-containing vacuoles may be sufficient to suppress the replication of *L. pneumophila* during *L. pneumophila*-caused pneumonia [59]. In summary, these studies indicate that itaconate has anti-viral as well as anti-bacterial functions by limiting both viral replication and bacterial growth.

## Conclusion

Accumulating evidence suggests that itaconate plays a potentially crucial role in orchestrating immune responses in the lungs and can exhibit complex immunoregulatory and anti-fibrotic functions. The discovery of naturally occurring isomers of itaconate, as well as a newly identified itaconate receptor, opens up a new and interesting avenue for therapeutic exploration. Thus, by virtue of its unique anti-inflammatory and antimicrobial properties, itaconate emerges as a promising therapeutic target for the treatment and management of respiratory conditions, such as asthma, fibrosis, bacterial, and viral infections.

## Abbreviations

4-OI: 4-octyl itaconate
ACOD-1: aconitate decarboxylase-1
AMs: airway macrophages
ATF3: activating transcription factor 3
COPD: chronic obstructive pulmonary disease
DI: dimethyl itaconate
GclmI: glutamate-cysteine ligase modifier subunit
HDM: house dust mite
HIF1α: hypoxia-inducible factor 1-alpha
Hmox1: haeme oxygenase 1
HSV: herpes simplex virus
IL: interleukin
ILC: innate lymphoid cells
IPF: idiopathic pulmonary fibrosis
Irg1: immune-responsive gene 1
KEAP1: Kelch-like ECH-associated protein 1
NFKB: nuclear factor kappa B
Nfkbiz: NFKB inhibitor zeta
Nqo1: NAD(P)H quinone dehydrogenase 1
OXGR1: oxoglutarate receptor 1
SDH: succinate dehydrogenase
TCA-cycle: tricarboxylic acid cycle
TH: T helper TH
TLRs: toll-like receptors
TNF: tumor necrosis factor.
